# Elbow arthroplasty: an Irish perspective

**DOI:** 10.1007/s11845-025-03960-1

**Published:** 2025-05-01

**Authors:** Nicolaas Leon Kotze, Diarmuid Molony, Olivia Flannery

**Affiliations:** 1 Connolly Hospital Blanchardstown, Dublin, Ireland; 2 Tallaght University Hospital, Dublin, Ireland; 3 Cappagh National Orthopaedic Hospital, Dublin, Ireland

**Keywords:** Distal humeral hemiarthroplasty, Ireland, Surgeon volume, Total elbow arthroplasty

## Abstract

**Background:**

Elbow arthroplasty (EA) aims to restore function and alleviate pain in the elbow joint. Research shows that a higher volume of EAs performed by surgeons and surgical centres correlates with decreased complications, reduced revision rates, and lower healthcare costs.

**Aims:**

This study aims to determine the current landscape of elbow arthroplasty in Ireland.

**Method:**

A retrospective cross-sectional study was conducted through surveys distributed to upper limb surgeons in Ireland who performed EAs from October 2022 to October 2024. The survey sought to gather data on the number of procedures completed in the 2-year window. Additionally, it probed surgeons’ intentions to continue performing these surgeries and their views on the necessary number of surgeons for adequate service provision in Ireland.

**Results:**

Nineteen surgeons participated, performing a total of 97 elbow arthroplasties over 2 years, which translates to a median of 4 procedures per surgeon (approximately 2 annually). The cohort included 32% distal humerus hemiarthroplasties (DHHs) and 68% total elbow arthroplasties (TEAs), with 60% classified as elective and 40% as trauma-related. All participants indicated a desire to continue performing these procedures.

**Conclusion:**

The low volume of elbow arthroplasty procedures in Ireland underscores the necessity for a national policy focused on enhancing surgical quality and patient outcomes. The insights gained from this data aim to stimulate discussions among elbow surgeons in Ireland, paving the way for effective policy implementation.

## Introduction

The primary objective of elbow arthroplasty is to restore joint function and alleviate pain in patients with elbow pathology. Total elbow arthroplasty (TEA) has traditionally been indicated for inflammatory arthropathies in low-demand individuals, particularly rheumatoid arthritis (RA). The advancement of medical therapies has meant that severe disease in this group is now rarer. The current indications for TEA have changed accordingly. These now encompass osteoarthritis, acute distal humerus trauma, non-union, tumour resection reconstruction, and articular damage due to haemarthrosis in patients with haemophilia and other blood diseases. Depending on the degree of constraint required and the integrity of the surrounding ligaments, TEA can be categorized as either linked or unlinked implants [1, 2].

TEA remains a technically challenging procedure on a small joint, often yielding worse outcomes compared to hip and knee arthroplasties. Reported complication rates range from 11 to 38%, including loosening, infection, wear, and ulnar neuropathy. Notably, better outcomes have been observed in patients undergoing TEA for RA and elderly patients sustaining acute distal humerus fractures, in contrast to those undergoing TEA for post-traumatic arthritis. Linked prostheses exhibit superior survival rates compared to unlinked counterparts, with 85.5% survival at 7.8 years compared to 74% at 12.3 years, respectively [3].

Distal humerus hemiarthroplasty (DHH) presents a viable alternative to TEA and open reduction internal fixation (ORIF) in treating various distal humerus pathologies. Indications for DHH can be classified into traumatic and non-traumatic categories, with unfixable distal humerus fractures being the most prevalent traumatic indication. DHH has also been utilized in cases of RA, non-union, osteomyelitis, and significant bone loss. Outcomes of DHH for non-traumatic indications have demonstrated superior results when compared to traumatic indications, yielding good to excellent outcomes in 76.5% of patients versus 67.4% for traumatic cases. In contrast, post-traumatic DHH reported fewer complications than non-traumatic DDH (27.6% vs. 50%) and preserved a greater arc of flexion–extension (98.3° vs. 61.8°) [4].

A systematic review comparing DHH to ORIF for intra-articular distal humerus fractures noted complication rates of 33% in the DHH cohort versus 38% in the ORIF group. Common complications following DHH included periprosthetic fracture, loosening, infection, and ulnar neuropathy. Despite lower complication rates in the DHH group, the overall complication rate remained significant [5].

Given the complexity, rarity, and relatively less favourable outcomes associated with elbow arthroplasties compared to hip and knee arthroplasties, questions surrounding the necessary experience for surgeons undertaking these procedures have arisen. A systematic review encompassing over 12,000 patients examined the correlation between TEA outcomes and surgeon or hospital volume. It concluded that lower surgical volumes correlated with increased revision rates, while complication rates remained comparable between surgeon volumes [6]. Further research focused on high-volume hospitals showed that those performing more than 22 TEAs annually demonstrated lower 90-day complication and revision rates, along with reduced healthcare costs [7].

Analysis of the British Joint Registry by the British Elbow and Shoulder Society (BESS) revealed that in 2016, a total of 401 TEAs were performed, contrasting sharply with 101,651 total hip arthroplasties in the same year. An assessment of surgeon volume revealed 141 centres and 170 consultants shared the responsibility for the 401 TEAs, averaging 2–3 TEAs per centre annually. Consequently, the BESS established guidelines aimed at consolidating TEA providers to enhance surgeon experience and facilitate the development of regional centres for these services [8].

Ireland lacks a joint registry for upper limb arthroplasty, making data regarding the current situation less accessible compared to other European countries [9]. The aim of this study was to determine the number of upper limb surgeons in Ireland who perform TEAs and the average number each surgeon performs annually.

## Methods

A retrospective cross-sectional study was designed utilizing a survey distributed to all upper limb specialist consultants in Ireland known to perform TEA or DHH. Surgeries performed from October 2022 to October 2024 were included, encompassing both primary and revision surgeries. The survey solicited the following information:

1. Total number of elbow arthroplasties performed throughout the consultant’s career.
2. Number of elbow arthroplasties performed within the specified time frame.
3. Breakdown of procedures into TEA/DHH and elective/trauma indications.
4. Interest in continuing to perform elbow arthroplasties.
5. Preference for performing elbow arthroplasties independently or with the collaboration of another consultant.
6. Opinion on the appropriate number of elbow arthroplasty surgeons required in Ireland.

## Results

Surveys were distributed to 19 identified surgeons, all of whom reported performing elbow arthroplasties. A total of 97 elbow arthroplasties were recorded, with a mean of 4.84, median of 4 and Standard Deviation of 2.33. Notably, there were 31 reported DHH procedures compared to 66 TEAs. Of the total cases, 58 were elective procedures, while 39 were trauma-related. A total of 15 revision surgeries were divided among 9 surgeons. Cumulatively, across their careers, the surgeons reported performing 443 elbow arthroplasties, with a mean of 22.63, median of 1, and standard deviation of 15.06.

All participating surgeons expressed the desire to continue performing elbow arthroplasties, with 74% (*n* = 14) preferring to do so independently, while 26% (*n* = 5) favoured having another consultant present.

**Fig. 1:**
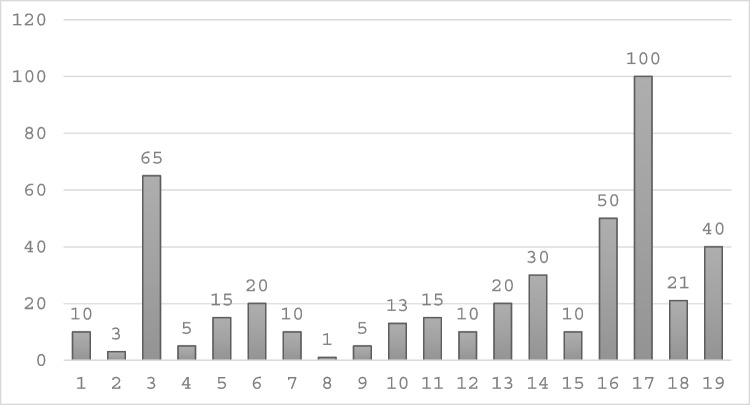
Total number of elbow arthroplasty performed by Irish upper limb surgeons during their career

**Fig. 2:**
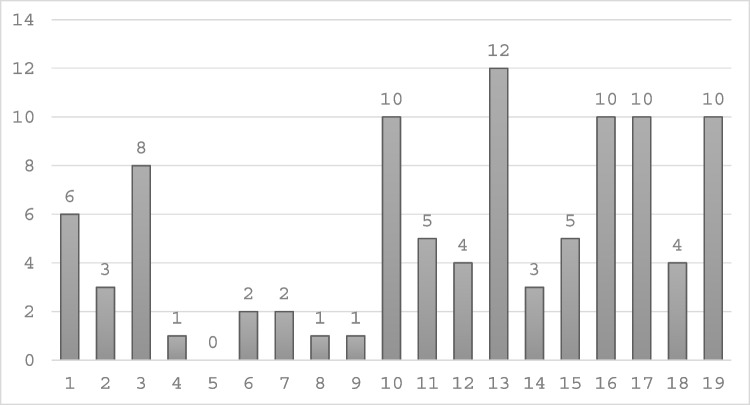
Total number of elbow arthroplasty performed by Irish upper limb surgeons between Oct 2022 and Oct 2024

**Fig. 3:**
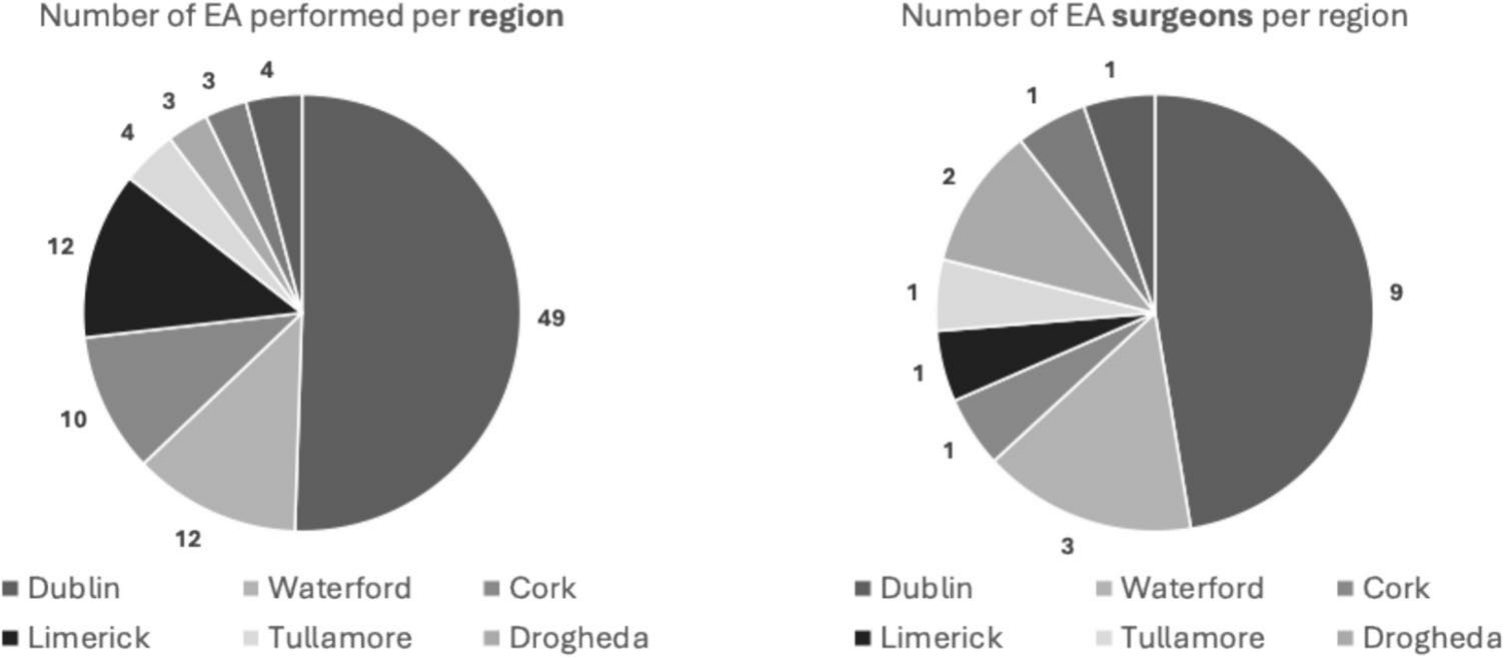
Number of EA performed per region and surgeons per region between Oct 2022 and Oct 2024

**Fig. 4:**
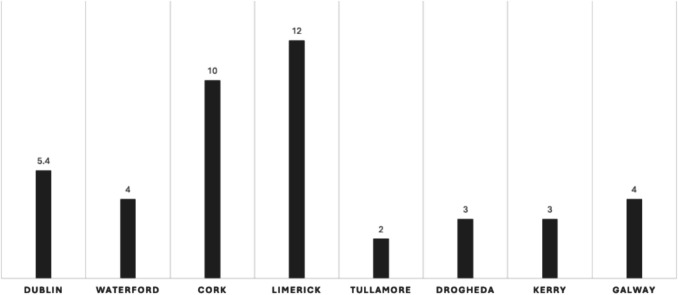
Average surgeries per surgeon per region performed by Irish upper limb surgeons between Oct 2022 and Oct 2024

**Fig. 5:**
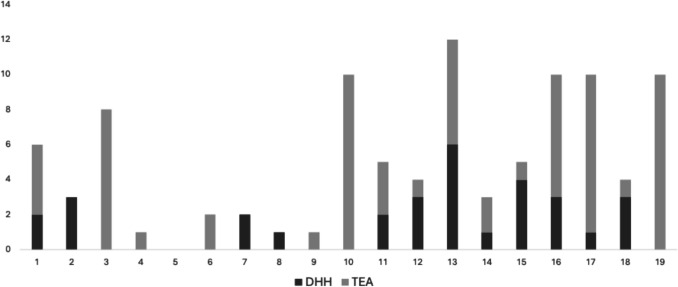
DHH vs TEA performed by Irish upper limb surgeons between Oct 2022 and Oct 2024

**Fig. 6:**
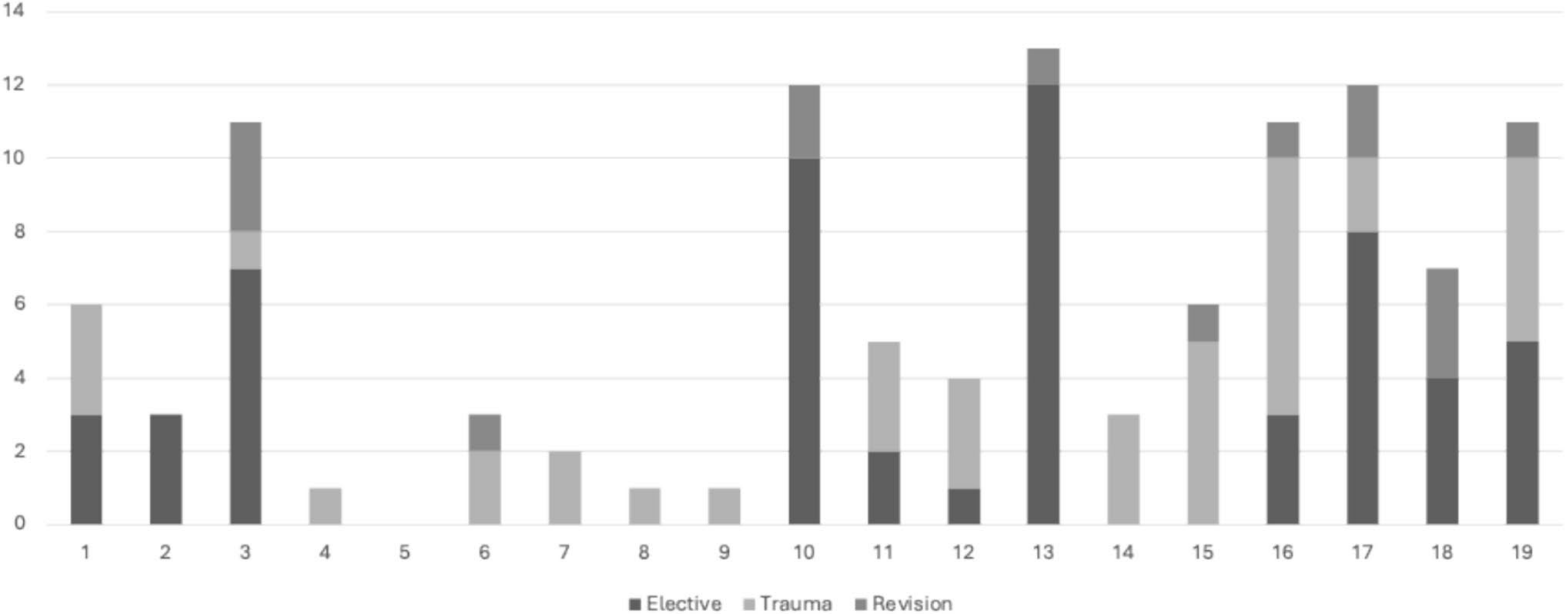
Trauma vs elective EA performed and revision surgery performed by Irish upper limb surgeons between Oct 2022 and Oct 2024

**Fig. 7:**
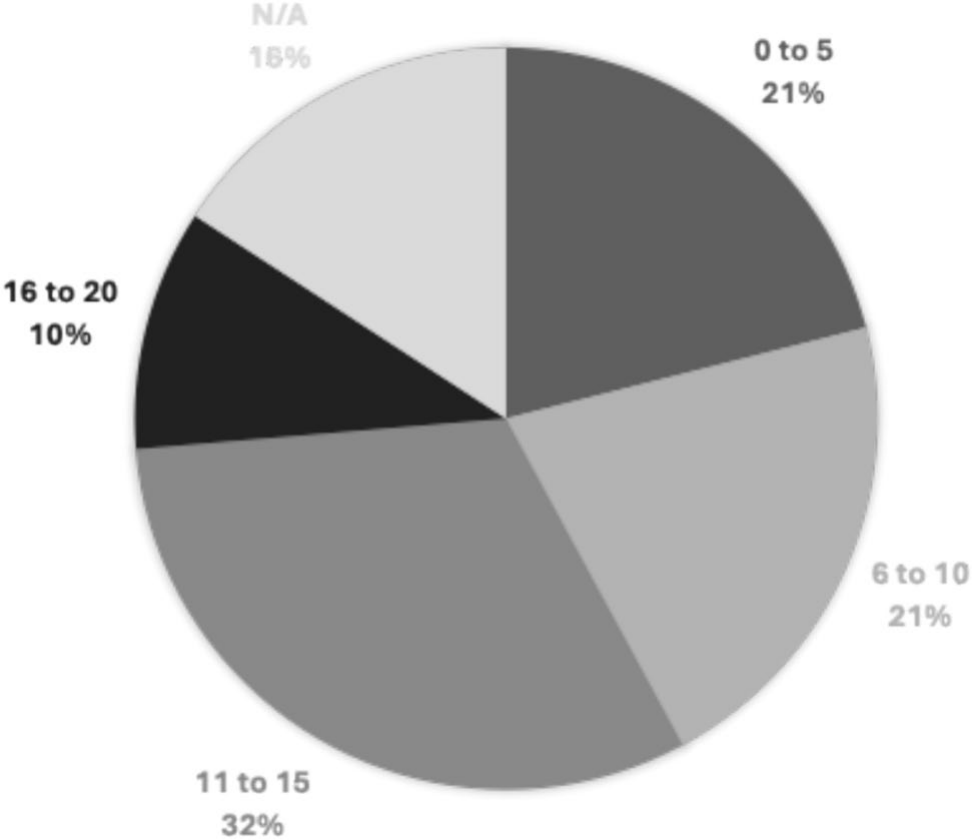
Opinion of Irish upper limb surgeons on number of EA surgeons Ireland should have

There was considerable variability in responses regarding the optimal number of elbow arthroplasty surgeons in Ireland. Specifically, 4 surgeons indicated that 5 or fewer surgeons would suffice, 4 suggested 5 to 10, another 4 believed 10 to 15 would be appropriate, and 4 recommended between 15 to 20. Three surgeons opted not to provide an opinion on this matter (Figs. 1, 2, 3, 4, 5, 6, 7).

## Discussion

Elbow arthroplasty has evolved significantly, with indications expanding beyond rheumatoid arthritis to include acute trauma, post-traumatic osteoarthritis, and non-union. Given the technical complexities associated with this procedure, particularly in comparison to hip, knee, and shoulder arthroplasties, it is imperative to recognize the importance of experienced surgeons and surgical centres in achieving optimal outcomes [1].

Evidence suggests that an increased surgical volume correlates with lower revision rates, although complication rates remain consistent across various volumes of practice [6]. Performing elbow arthroplasties in high-volume centres has shown to decrease complications and revisions while reducing overall healthcare costs [7].

The data from this study illustrate the relative rarity of elbow arthroplasties in Ireland, with only 97 procedures performed over a 24-month period across 18 centres, averaging 48.5 procedures annually equating to 2.69 procedures per centre per year. In contrast, the Irish National Orthopaedic Register reported 3723 hip arthroplasties and 2871 knee arthroplasties over a 56-month period, highlighting the disparity in surgical volume within orthopaedic practices in Ireland [9].

When considering individual surgeons, the sampled cohort of 19 surgeons performed only an average of 2.55 elbow arthroplasties per surgeon annually. The willingness of these surgeons to continue performing the procedure, coupled with their split opinions regarding the necessary number of elbow arthroplasty surgeons in Ireland, underscores the need for clarification on service provisions in this niche area of orthopaedics.

In light of the data collected, it is reasonable to advocate for the establishment of a national policy on elbow arthroplasty akin to that recommended by the British Shoulder and Elbow Society. However, further work is required to draft, approve, and implement such a policy. This initiative is essential for better patient care and outcomes in Ireland, particularly for those undergoing these specialized procedures in the future.

Due to the risk of recall bias, the data of this study was compared to the data from the UK study. In 2016, there was 401 TEA performed in the UK with an estimated population size 65,648,000 [10]. This correlates to 1 TEA for every 163,710 people in the UK. The Irish population during the 2022 census was 5,139,149 [11]. According to the data collected in this study, there is an annual average of 33 TEA performed in Ireland. This would mean there was 1 TEA performed for every 155,731 people in Ireland.

Although the data from this study seems consistent with findings from the UK, the study design includes an inherent recall bias. To improve the reliability of the data, we will conduct a follow-up study that will collect prospective data from January 2025 to December 2026. Furthermore, it is expected that there will be a transition toward more regional management of elbow arthroplasties.

## Data Availability

Data is available from the primary author upon request.

## References

[1] Sanchez-Sotelo J (2011) Total elbow arthroplasty. Open Orthop J 5:115–123. 10.2174/1874325001105010115 21584200 10.2174/1874325001105010115 PMC3093740

[2] Samdanis V, Manoharan G, Jordan RW et al (2020) Indications and outcome in total elbow arthroplasty: a systematic review. Should Elb 12:353–361. 10.1177/1758573219873001 10.1177/1758573219873001 PMC7545529 33093874

[3] Welsink CL, Lambers KTA, van Deurzen DFP, Eygendaal D, van den Bekerom MPJ (2017) Total elbow arthroplasty: a systematic review. JBJS Rev 5:e4. 10.2106/jbjs.Rvw.16.00089 28696952 10.2106/JBJS.RVW.16.00089

[4] Dunn J, Kusnezov N, Pirela-Cruz M (2014) Distal humeral hemiarthroplasty: indications, results, and complications. A systematic review. Hand (N Y) 9:406–412. 10.1007/s11552-014-9681-3 25414601 10.1007/s11552-014-9681-3 PMC4235903

[5] Nielsen AF, Al-Hamdani A, Rasmussen JV, Olsen BS (2022) Elbow hemiarthroplasty vs. open reduction internal fixation for acute Arbeitsgemeinschaft für Osteosynthesefragen/Orthopaedic Trauma Association (AO/OTA) type 13C fractures-a systematic review. JSES Int 6:713–722. 10.1016/j.jseint.2022.06.002 36081704 10.1016/j.jseint.2022.06.002 PMC9446201

[6] Prkić A, Vermeulen NP, Kooistra BW et al (2023) Is there a relationship between surgical volume and outcome for total elbow arthroplasty? A systematic review. EFORT Open Rev 8:45–51. 10.1530/eor-22-0087 36705616 10.1530/EOR-22-0087 PMC9969007

[7] Poff C, Kunkle B, Li X et al (2022) Assessing the hospital volume-outcome relationship in total elbow arthroplasty. J Shoulder Elbow Surg 31:367–374. 10.1016/j.jse.2021.08.025 10.1016/j.jse.2021.08.025 34592413

[8] Society BSaE (2018) British Shoulder and Elbow Society National Guidelines. https://bess.ac.uk/national-guidelines/##all_0-1420-wpfd-surgical-procedure-guidelines . Accessed 8 Nov 2024

[9] National Office of Clinical Audit (1 Dec 2014–31 July 2019) Irish National Orthopaedic Register First Report. https://www.noca.ie/documents/irish-national-orthopaedic-register-first-report/

[10] Office for National Statistics (2017) Population estimates for the UK, England and Wales, Scotland and Northern Ireland: mid 2016. https://www.ons.gov.uk/peoplepopulationandcommunity/populationandmigration/populationestimates/bulletins/annualmidyearpopulationestimates/mid2016

[11] Central Statistics office (2022) Cencus of Population. https://www.cso.ie/en/statistics/population/censusofpopulation2022/censusofpopulation2022-summaryresults/

